# Shared Sociodemographic Risk Factors for Neurocognitive Dysfunction in Children With Cancer and Blood Disorders

**DOI:** 10.1002/pbc.32131

**Published:** 2025-10-27

**Authors:** Claire E. F. Miller, Jamie Neiman Luehring, Elizabeth B. Molina Kuna, Shweta Bhatia, Adam L. Green, Bruce E. Compas

**Affiliations:** 1 Division of Pediatric Hematology, Oncology, and Bone Marrow Transplant, Department of Pediatrics, Primary Children’s Hospital, University of Utah, Salt Lake City, Utah, USA; 2 Center For Cancer and Blood Disorders, Children’s Hospital Colorado, Aurora, Colorado, USA; 3 Population Health Shared Resource, University of Colorado Cancer Center, Aurora, Colorado, USA; 4 Department of Pediatrics, University of Colorado School of Medicine, Aurora, Colorado, USA; 5 Department of Psychology and Human Development, Vanderbilt University, Nashville, Tennessee, USA

**Keywords:** neurocognitive function, pediatric hematology/oncology, psychosocial, social determinants of health

## Abstract

**Background:**

To investigate the role of social determinants of health (SDoH) in neurocognitive functioning among children with acute lymphoblastic leukemia (ALL), central nervous system (CNS) tumors, and sickle cell disease (SCD).

**Procedure:**

Fifty-eight child-caregiver dyads participated. SDoH included components of socioeconomic status (SES; family income, caregiver education, and insurance type), race/ethnicity, caregiver marital status, and neighborhood-level deprivation. Neurocognitive functioning was measured using the NIH Toolbox Cognition Battery (NIHTB-CB) Fluid Cognition subtests.

**Results:**

There were no differences in SDoH between the three diagnosis groups other than expected differences in race/ethnicity (*χ*^2^ (4, *n* = 51) = 46.1, *p* < 0.001). Bivariate analyses found a significant association between family income and working memory (*r* = 0.31, *p* < 0.05), processing speed (*r* = 0.29, *p* < 0.05), and fluid cognition composite scores (*r* = 0.29, *p* < 0.05). No other SDoH were associated with neurocognitive functioning. Family income remained significantly associated with performance on working memory (*β* = 0.35, *p* < 0.05), processing speed (*β* = 0.33, *p* < 0.05), and fluid cognition composite scores (*β* = 0.29, *p* < 0.05) after controlling for diagnosis, age, and sex.

**Conclusions:**

Lower family income emerged as an important element of SES associated with poorer neurocognitive functioning in children treated for cancer and blood disorders. Further research is needed to determine how family income alters neurocognitive functioning, whether by altering the timing of emergence or the magnitude of deficits. Future studies should investigate mechanisms linking low income to neurocognitive performance to inform interventions to bolster neurocognitive development.

## 1 | Introduction

Therapies for pediatric cancers and blood disorders are improving rapidly, allowing children to survive previously fatal diseases [1–3]. As children survive longer, unintended consequences of the diseases and treatments have become more apparent, with neurocognitive dysfunction being particularly impactful in its effect on the daily lives of survivors [4, 5]. Children at high risk for dysfunction include those with leukemia, central nervous system (CNS) tumors, and sickle cell disease (SCD) [6–11]. Our group previously conducted a cross-sectional evaluation of neurocognitive functioning in these at-risk groups and used a common metric (i.e., NIH Toolbox Cognition Battery) to facilitate direct comparison between groups [12]. Direct comparison revealed disease and domain-specific patterns of neurocognitive dysfunction, with 27 children with acute lymphoblastic leukemia (ALL) demonstrating significantly worse performance compared to normative data on four of the five fluid cognition subtests administered, 11 participants with CNS tumors demonstrating large, negative effects in processing speed and inhibitory control and attention, and 15 participants with SCD largely performing in the average range with cognitive flexibility being the only domain negatively affected [12]. While each of these populations is exposed to unique disease and treatment-related risk factors [8, 13–16], the present study seeks to extend this work by examining shared risk factors (i.e., social determinants of health, SDoH) among children with cancer and blood disorders that confer additional risk for neurocognitive dysfunction.

The impact of SDoH on neurocognitive functioning is understudied in pediatric hematology oncology compared to the literature on typically developing children [17], where socioeconomic status (SES) is the determinant most commonly measured. While SDoH encompass a vast range of conditions in the environment that affect health outcomes (e.g., transportation, access to nutritious foods) [18], SES more narrowly encompasses family income, education, occupation, and neighborhood resources that similarly affect health outcomes [19]. In typically developing children there is robust data linking low SES and neurocognitive dysfunction (particularly in the domains of language and executive function), higher rates of developmental delay, and lower school achievement [20–23]. In typically developing children, differences in executive function across SES gradients emerge as early as infancy and persist into adulthood [24]. Language follows a similar, if not more profound trajectory; a defining study by Hart and Risley demonstrated that 3-year-old children from professional families had twice the vocabulary, on average, compared to children in families on welfare [23–25].

Factors making up SES (i.e., income, parental education, occupation, and neighborhood resources) are intimately related but relate to children’s brain structure and cognitive development in unique ways [26–28]. For example, in a sample of 1099 typically developing children, family income was logarithmically associated with brain surface area while parental education was linearly associated [26]. In addition, a study of 3892 typically developing children found that family income had medium effects on fluid cognition, while parental education and neighborhood deprivation each had small effects [28]. Given the well-established (albeit complex) relationship between elements of SES and neurocognitive functioning, closer examination of SES might provide a source for important modifiable risk factors of neurocognitive dysfunction in children with cancer and blood disorders [29–31]. Examining executive functioning is particularly important, since it is known to be impacted by SES in typically developing children and impacted by disease and treatment-related variables in children with cancer and blood disorders [6–10, 23, 24]. Among children with cancer and blood disorders, a limited number of studies have similarly found a relationship between higher SES and better cognitive outcomes and school performance [32–37]; however, the distinctive factors making up SES have not been systematically examined in this population. Developing a more robust understanding of the unique contributions that family income, parental education, neighborhood context, and other SDoH have on cognitive outcomes is paramount, so that targets can be identified and intervened upon.

The aim of the present study is to investigate the role of selected SDoH in children’s neurocognitive performance on the NIH Toolbox Cognition Battery (NIHTB-CB) among children with ALL, CNS tumors, and SCD. First, we hypothesize that participants with SCD will have higher rates of public insurance, lower income, lower caregiver education, and live in neighborhoods with higher deprivation because of the confounding effects of Black race and systemic racism [38]. We hypothesize these SDoH will not differ between children with ALL and CNS tumors. Second, we hypothesize that proximal indicators of SES, namely, family income and caregiver education will best predict neurocognitive performance, rather than proxy (e.g., insurance status) or more distal measures (i.e., neighborhood-level deprivation) of SES.

## 2 | Methods

A cross-sectional study design was employed to enroll participants ages 7–12 years with a diagnosis of ALL, CNS tumor, or SCD and measured aspects of neurocognitive function using the NIHTB-CB. The study protocol was approved by the Colorado Multiple Institutional Review Board. Parents and caregivers provided informed consent and participants provided assent prior to study participation.

### 2.1 | Participants

English and Spanish-speaking patients between the ages of 7–12 years with pre-B ALL, CNS tumors, or SCD (i.e., hemoglobin SS or S*β*^0^) were invited to participate in the present study. Strict inclusion/exclusion criteria were applied to make the cohort as homogeneous as possible. Participants were eligible for participation if they did not have a pre-existing neurological disorder (e.g., neurofibromatosis) or known intellectual disability. Among children with ALL and CNS tumors, participants were eligible for participation if they had completed therapy within the past 18 months; since SCD is most often diagnosed at birth, this criterion was not applicable. Participants with CNS tumors must have received a diagnosis via biopsy or tumor markers and were not eligible for participation if their tumor was diagnosed with imaging alone. Participants with ALL were excluded if they had a history of symptomatic hyperleukocytosis. Finally, participants with SCD were included if they took hydroxyurea, but were ineligible if they had a history of overt stroke, received chronic exchange transfusions, or had undergone bone marrow transplantation.

### 2.2 | Procedures

#### 2.2.1 | Neurocognitive Battery

Neurocognitive functioning was measured using the NIHTB-CB, version 2 [39]. The NIHTB-CB is a widely used measure of neurocognitive functioning in clinical research given its inclusive age range, ease of administration, sound psychometrics, and inclusion of multiple subtests of executive functioning [40, 41]. Given evidence of difficulties in attention, processing speed, and executive functioning in these populations, the Fluid Cognition subtests were administered [7, 9, 10, 16], including three subtests measuring executive function (i.e., List Sort Working Memory, Flanker Inhibitory Control and Attention, Dimensional Change Card Sort), the Pattern Comparison Processing Speed subtest, and the Picture Sequence Memory subtest. These five subtests can be taken together to yield a Fluid Cognition Composite Score. Crystalized cognition subtests were not administered given the desire to prioritize fluid cognition measurement, time constraints, and evidence that participants outperform normative data on NIHTB-CB crystalized cognition subtests [42]. Results from the NIHTB-CB are presented as age-adjusted standard scores with a mean of 100 and standard deviation of 15.

#### 2.2.2 | Sociodemographic Measures

Caregivers completed demographic questionnaires via iPad, providing information about their child’s race, ethnicity, and insurance. In addition, caregivers provided their own highest level of education attained, family income, and marital status. Caregiver education was transformed from categorical (e.g., “some high school,” “high school diploma”) to ordinal by assigning number of years of education to each category (e.g., “high school diploma” = 12, “college degree” = 16). Similarly, caregivers were given ranges to report their income (e.g., <$20,000, $20,000–$29,999); income midpoints were calculated so that income could be treated as a continuous variable rather than ordinal. Marital status was collapsed into dichotomous groups (i.e., single or partnered) such that “single and never married,” “separated,” “divorced,” and “widowed” were coded as single and “In a relationship/living with someone” and “married” were coded as partnered.

Participant zip code was obtained via chart review and used to assign a Social Deprivation Index (SDI) to provide insight into participants’ neighborhood environments and a neighborhood-level SDoH measure. The SDI is a composite measure of area level deprivation that considers seven demographic characteristics from the American Community Survey, including the proportion of people living in poverty, having less than 12 years of education, living in single-parent households, living in rented housing units, living in overcrowded housing units, households without a car, and non-employed adults under 65 years of age [43]. Zip codes were used to assign the 2019 SDI score for each participant, with higher scores indicating higher levels of deprivation. Once SDI was calculated, zip codes were removed from the dataset for de-identification purposes. Remaining details on the recruitment strategies and additional study procedures are published in Fraley et al. [12].

#### 2.2.3 | Statistical Analysis

To compare selected SDoH between disease groups, a Chi Square test was employed for categorical variables (e.g., race/ethnicity) and one-way analysis of variance (ANOVA) was used for continuous variables (e.g., income). In analyses of the group as a whole, SDoH categories were either combined (e.g., marital status, as above) or removed from analysis with *n* < 5 (e.g., Native American/Alaska native *n* = 1, mixed race *n* = 2).

To investigate the role of SDoH on NIHTB-CB performance, a bivariate analysis was performed with Pearson’s correlations for continuous SDoH variables. Next, a one-way analysis of covariance (ANCOVA) was performed for categorical SDoH (i.e., race/ethnicity, marital status, employment) while controlling for disease group. If the ANCOVA reached statistical significance, pair-wise comparison *t*-tests were performed. Finally, multiple linear regression (MLR) was performed to assess the association of SDoH with NIHTB-CB performance in the context of relevant clinical and demographic variables. SDoH were included in MLR models if they reached statistical significance in bivariate analyses. Clinical and demographic variables included diagnosis, age at assessment, and gender. Age at diagnosis was not included given that participants with SCD were diagnosed at birth, whereas patients with ALL and CNS tumors were diagnosed later in life.

## 3 | Results

### 3.1 | Demographics

Fifty-eight participants and their caregivers participated in the study (27 with ALL, 14 with CNS tumors, and 17 with SCD) between May 2021 and November 2022; this includes five additional participants who completed study activities following the initial publication on neurocognitive performance in this cohort [12]. Children with ALL (37% female) were on average 5.85 years old at the time of diagnosis (SD = 1.96) and were on average 9.15 years old at the time of testing (SD = 1.70). No participants with ALL had CNS involvement. The mean number of intrathecal doses of chemotherapy was 20 (SD = 3). Eight participants (29.6%) were treated with high-dose methotrexate (the remaining with escalating doses of methotrexate), there were no documented cases of methotrexate neurotoxicity, and one participant had a prolonged (i.e., greater than 72 hours) intensive care unit admission. Children with CNS tumors (29% female) were on average 8.57 years old at the time of diagnosis (SD = 1.65) and were 9.79 years old at the time of testing (SD = 1.48). Many tumor types were included, with medulloblastoma and embryonal tumors comprising the largest group (*n* = 4, 28.6%), followed by germ cell tumors (*n* = 3, 21.4%) and low-grade gliomas (*n* = 3, 21.4%). Tumor location was equally divided between the posterior fossa and supratentorial region. Twelve children were treated with surgery (85.7%), nine received chemotherapy (64.3%), and twelve received radiation (85.7%) as part of their therapy. Nearly all children with CNS tumors presented with hydrocephalus (*n* = 12, 85.7%). Children with SCD (65% female) were on average 8.29 years old at the time of testing (SD = 1.76). Hemoglobin SS was the most common genotype represented (*n* = 16, 94.1%), with one participant having S*β*^0^ genotype (5.9%). Four participants had a conditional result on the most recent transcranial doppler (23.5%). Mean hemoglobin A at time of testing was 8.7 g/dL (SD = 3.0), mean hemoglobin F was 22.0 g/dL (SD = 11.3), and mean number of hospitalizations was 5 (SD = 4.73). In the sample as a whole, the majority of caregiver respondents were mothers (*n* = 45, 80.4%), followed by fathers (*n* = 11, 19.3%), with one grandmother providing demographic and SDoH data. Caregivers did not provide data for *n* = 4 child race/ethnicity, *n* = 1 insurance, *n* = 2 family income, and *n* = 1 caregiver education. Additional participant demographic and clinical information has been previously published [12].

When comparing SES and other social determinants between the three disease groups, as expected, there were significant differences detected for race/ethnicity, with more Black, non-Hispanic patients with SCD (*χ*^2^ (4, *n* = 51) = 46.1, *p* < 0.001; Table 1). However, there were no significant differences seen for insurance type, marital status, family income, caregiver education, or SDI between disease groups.

### 3.2 | Bivariate Analyses

To assess for multicollinearity, correlations were calculated to measure associations between continuous variables (Table 2). Caregiver income and education were positively correlated with one another (*r* = 0.51, *p* < 0.01), corresponding with higher income among more highly educated families. SDI was negatively correlated with both income (*r* = −0.49, *p* < 0.01) and education (*r* = −0.44, *p* < 0.05), reflecting families with lower income and education living in neighborhoods with higher levels of deprivation.

Next, associations between SDoH and scores on the NIHTB-CB scales were calculated using Pearson’s correlation for continuous SDoH and ANCOVA for categorical SDoH (Tables 2 and 3). Family income correlated significantly with performance on working memory (*r* = 0.31, *p* = 0.027) and processing speed tasks (*r* = 0.29, *p* = 0.040), in addition to the Fluid Cognition Composite score (*r* = 0.29, *p* = 0.040; Table 2). Family income was not significantly associated with episodic memory, inhibitory control and attention, or cognitive flexibility. Caregiver education, SDI (Table 2), race/ethnicity, insurance type, and marital status were not associated with the child’s performance on the NIHTB-CB (Table 3).

### 3.3 | Multiple linear regression

Finally, MLR was used to investigate the association of SDoH with children’s cognitive functioning in the context of diagnosis, current age, and gender (Table 4). Since family income was the only variable significantly associated with cognitive functioning at the bivariate level, it was the sole SDoH included in MLR. Models for processing speed (Δ*R*^2^ = 0.19, *p* < 0.05) and inhibitory control and attention (Δ*R*^2^ = 0.27, *p* < 0.01) reached statistical significance, accounting for 18.9% and 26.6% of the variability in scores, respectively. Diagnosis (*β* = 0.29, *p* < 0.05) and family income (*β* = 0.33, *p* < 0.05) were significant predictors of processing speed scores, with children with SCD and families with higher income having higher scores. Age (*β* = −0.46, *p* < 0.001) and gender (*β* = −0.27, *p* < 0.05) were significant predictors of performance on the inhibitory control and attention subtest, with younger participants and boys having higher scores. Though the models for working memory and the fluid cognition composite did not reach statistical significance for goodness of fit, family income was significantly associated with these scores (*β* = 0.35, *p* < 0.05; *β* = 0.29, *p* < 0.05, respectively). Models accounting for caregiver education and SDI can be found in Table S1. Individual contributions of caregiver education and SDI did not reach statistical significance in these models.

## 4 | Discussion

This study examines associations between SDoH, with particular focus on components of SES, and cognitive functioning in at-risk children treated for cancer and blood disorders. Despite expected differences in race/ethnicity between groups of children with ALL, CNS tumors, and SCD, there were no differences between groups based on insurance type, caregiver marital status, family income, caregiver education, or neighborhood deprivation as measured by the SDI, contrary to the first hypothesis (Table 1). Importantly, there was little missing caregiver-reported sociodemographic data. Although income, education, and SDI were highly correlated with each other, family income was the only social determinant that was associated with cognitive scores in bivariate and regression analyses, such that children from families with higher incomes had higher scores in working memory, processing speed, and fluid cognition composite scores (Tables 2 and 3 and Table S1). Caregiver education, neighborhood deprivation, race/ethnicity, insurance type, and caregiver marital status were not predictive of neurocognitive functioning.

These findings align with those from much larger studies in typically developing children. In the ABCD study, a 10-year longitudinal multi-site study of 3892 typically developing children, family income, parent education, and neighborhood deprivation were similarly found to be significantly correlated with one another; in this instance, each had a main effect on cognitive performance measured by the NIHTB-CB. However, taken together in MLR, parent education and area deprivation were no longer significant, and instead family income remained predictive of fluid cognition [28]. Additional studies demonstrate the unique contribution of family income and caregiver education on brain structure. For example, the multisite Pediatric Imaging, Neurocognition, and Genetics (PING) study demonstrated that, among 1099 typically developing children, parental education and family income each had a main effect on brain surface area. Parental education had a positive, linear association with cortical surface area and income had a logarithmic one, meaning that small increases in income among lower SES families had greater influence on surface area while the same increases in income among higher SES families resulted in smaller increases in surface area [26]. When both education and income were included together in a model, however, only family income accounted for unique variance in cortical surface area [26].

It is noteworthy that race/ethnicity, patient insurance, caregiver marital status, caregiver education, *and* SDI did not significantly correlate with cognitive functioning, suggesting that family income may have a unique influence on the specific domains of working memory and processing speed in children with cancer and blood disorders, even within the context of disease and treatment-related risk factors. Income may have a particularly robust influence on aspects of cognitive functioning since it signifies access to material resources, including nutritious food, reliable childcare, books in the home (i.e., cognitive stimulation), and the ability to live in neighborhoods that allow for quality education, less crime, less toxic environmental exposures, and safe outdoor physical activity [24, 27, 44]. Additionally, brain regions underlying executive functioning (e.g., working memory) undergo a prolonged period of development, so are more susceptible to longstanding environmental influences from factors like family income [24]. Future studies should examine material resources associated with income, since provision of these resources may serve as a target of intervention to support cognitive development.

There are several notable limitations in the current study. First, the sample sizes were small, especially when split into disease and SDoH groups. Thus, the sample did not lend itself to moderation analyses to examine associations of SDoH with cognitive functioning within specific disease groups. It is possible that each disease group is uniquely affected by specific SDoH; future studies should seek to enroll sociodemographically diverse samples to carefully examine this possibility. Second, it is possible that certain disadvantaged groups were inadvertently excluded from study participation, making the current sample less diverse. For example, at least one Spanish-speaking mother was unable to complete the demographic questionnaire due to illiteracy, additional Spanish interpreters being unavailable, and transportation access. Moreover, strict inclusion/exclusion criteria in the SCD cohort likely limited not only the disease severity of the cohort, but also the socioeconomic diversity of the sample. In addition, Children’s Hospital Colorado serves a wide multi-state catchment area, and it is possible that disadvantaged patients living farther from the hospital were lost to follow up because of difficulty returning to the treatment facility, particularly during the COVID-19 pandemic. Finally, the SDI was created based on participants’ zip codes at the time of study participation, not at time of diagnosis. Knowing that up to one third of patients move during their cancer treatment [45], this distinction is important, since the child’s current SDI may not be reflective of the neighborhood environment they were exposed to for the majority of their childhood.

In addition to enrolling sociodemographically diverse cohorts, future studies should expand their focus beyond components of SES to examine other SDoH. Furthermore, future studies should incorporate longitudinal designs to investigate whether family income, its associated material resources, or other SDoH alter the trajectory of neurocognitive dysfunction in timing or magnitude. Longitudinal studies could also establish temporal precedence, thus allowing for mediation analyses to untangle the complex mechanisms linking SDoH like family income with neurocognitive outcomes. Having a more nuanced understanding of these mechanisms would highlight potential targets of intervention to support neurocognitive functioning in children with cancer and blood disorders.

## Supplementary Material

Supplemental Table S1

Supporting Information

Additional supporting information can be found online in the Supporting Information section.

## Figures and Tables

**TABLE 1:** Descriptive statistics of patient-reported social determinants of health in participants with leukemia, CNS tumors, and sickle cell disease

		Disease Type			
	Total N = 58 N (%)	ALL N = 27 N (%)	CNS Tumor N = 14 N (%)	SCD N = 17 N (%)	*p*-value
**Race/Ethnicity**					<0.001
White NH	22 (37.9)	15 (55.6)	7 (50.0)	0	
Hispanic	15 (25.9)	9 (33.3)	5 (35.7)	1 (5.9)	
Black NH	14 (24.1)	1 (3.7)	0	13 (76.5)	
**Insurance Type**					0.148
Public	24 (47.1)	10 (43.5)	4 (30.8)	10 (66.7)	
Private	27 (52.9)	13 (56.5)	9 (69.2)	5 (33.3)	
**Marital status**					0.309
Single	11 (19.0)	3 (11.1)	3 (21.4)	5 (29.4)	
Partnered	47 (81.0)	24 (88.9)	11 (78.6)	12 (70.6)	
	M (SD)	M (SD)	M (SD)	M (SD)	
**Family Income**	$64 732 ($30 513)	$70 096 ($30 907)	$60 192 ($30 421)	$60 000 ($30 439)	0.481
**Caregiver Education (years)**	15.5 (3.0)	15.0 (3.2)	15.6 (2.5)	16.1 (3.1)	0.634
**Social Deprivation Index**	36.5 (27.1)	35.3 (28.9)	29.6 (22.9)	43.8 (27.4)	0.343

**TABLE 2:** Pearson correlations between continuous SDoH and NIHTB-CB scores

	1	2	3
**1. Family Income**	--		
**2. Caregiver Education**	0.514**	--	
**3. SDI**	−0.490**	−0.436*	--
**4. Working Memory**	0.307*	0.199	−0.181
**5. Processing Speed**	0.285*	0.231	−0.082
**6. Episodic Memory**	0.094	0.170	0.023
**7. Inhibitory Control & Attention**	0.098	−0.091	−0.081
**8. Cognitive Flexibility**	−0.041	0.107	−0.047
**9. Fluid Composite**	0.288*	0.216	−0.098

* *p* < 0.05

** *p* < 0.01

Note: SDI = Social Deprivation Index

**TABLE 3:** Associations of social determinants of health on NIH Toolbox Cognition Battery scores after controlling for disease

	NIHTB-CB Scores					

	Working Memory	Processing Speed	Episodic memory	Inhibitory Control & Attention	Cognitive Flexibility	Fluid Composite

	M (SD)					

**Race/Ethnicity**						
White Non-Hispanic	99.3 (11.2)	84.0 (21.2)	98.8 (14.5)	89.5 (11.8)	94.7 (11.2)	88.6 (14.1)
Hispanic	90.3 (13.9)	81.5 (19.7)	99.5 (14.6)	93.3 (15.0)	90.9 (15.2)	85.2 (14.8)
Black Non-Hispanic	97.1 (11.3)	94.4 (22.2)	90.7 (9.5)	94.6 (13.9)	91.7 (17.0)	89.4 (13.6)

**Insurance**						
Public	93 (11.2)	85 (18.5)	96 (14.1)	93 (13.9)	96 (13.1)	87 (13.2)
Private	97 (12.2)	87 (22.5)	99 (14.0)	93 (11.9)	93 (14.4)	89 (14.5)

**Marital Status**						
Single	92.4 (16.7)	87.5 (18.1)	97.0 (10.7)	88.7 (13.7)	89.9 (10.0)	84.7 (11.2)
Partnered	96.6 (12.3)	87.7 (21.4)	97.6 (14.8)	93.5 (12.8)	94.0 (14.1)	89.6 (14.6)

* *p* < 0.05

**TABLE 4:** Multiple Linear Regression Analyses Predicting Cognitive Performance

	NIHTB-CB Scores											

	Working Memory		Processing Speed		Episodic memory		Inhibitory Control & Attention		Cognitive flexibility		Fluid Cognition Composite	

	*β*	*ΔR^2^*	*β*	*ΔR^2^*	*β*	*ΔR^2^*	*β*	*ΔR^2^*	*β*	*ΔR^2^*	*β*	*ΔR^2^*

Model 1.		0.144		0.189*		0.038		0.266**		0.004		0.121
Diagnosis	0.176		0.290*		−0.164		−0.046		−0.015		0.118	
Age	0.194		0.200		−0.073		−0.462***		0.035		−0.022	
Gender	0.011		−0.114		−0.073		−0.268*		0.025		−0.149	
Family Income	0.347*		0.333*		0.051		0.041		−0.039		0.293*	

* *p* < 0.05

** *p* < 0.01

*** *p* ≤ 0.001

## Data Availability

The data that support the findings of this study are available from the corresponding author upon reasonable request.

## Abbreviations

ALL: acute lymphoblastic leukemia
ANCOVA: analysis of covariance
ANOVA: analysis of variance
CNS: central nervous system
FSIQ: Full Scale Intelligence Quotient
NIHTB-CB: National Institutes of Health Toolbox Cognition Battery
SCD: sickle cell disease
SDI: Social Deprivation Index
SDoH: social determinants of health
SES: socioeconomic statu
