# Supplementary material for: Shared Sociodemographic Risk Factors for Neurocognitive Dysfunction in Children With Cancer and Blood Disorders
Source: Pediatr Blood Cancer. Author manuscript; Available in PMC 2026 Feb 10. (PMC12890220; doi:10.1002/pbc.32131)
Supplement: Supplemental Table S1 [file NIHMS2120513-supplement-Supplemental_Table_S1.docx]

**SUPPLEMENTAL TABLE S1** Supplemental Multiple Linear Regression Analyses Predicting Cognitive Performance

|  | **NIHTB-CB Scores** | | | | | |
| --- | --- | --- | --- | --- | --- | --- |
|  | **Working Memory** | **Processing Speed** | **Episodic memory** | **Inhibitory Control & Attention** | **Cognitive flexibility** | **Fluid Cognition Composite** |
|  | *β ΔR^2^* | *β ΔR^2^* | *β ΔR^2^* | *β ΔR^2^* | *β ΔR^2^* | *β ΔR^2^* |
| Model 2.  Diagnosis  Age  Gender  Caregiver Education | 0.133  0.057  0.281†  -0.132  0.160 | 0.166  0.021  0.303†  -0.145  0.196 | 0.118  -0.158  -0.014  -0.232  0.184 | 0.341**  -0.209  -0.524***  -0.232  -0.034 | 0.034  -0.142  -0.090  -0.018  0.140 | 0.140  -0.142  0.024  -0.245  0.224 |
| Model 3.  Diagnosis  Age  Gender  SDI | 0.071  0.148  0.154  -0.058  -0.019 | 0.119  0.256†  0.208  -0.162  -0.105 | 0.419  -0.163  -0.054  -0.086  0.027 | 0.304*** -0.015  -0.512***  -0.261*  -0.143 | 0.003  -0.015  -0.010  -0.022  -0.049 | 0.068  0.071  0.004  -0.233  -0.127 |

†*p* < 0.10, **p* < 0.05, ***p* < 0.01, ****p* ≤ 0.001

Note: NIHTB-CB = NIH Toolbox Cognition Battery
